# Supplementary figures and images for: Paraburkholderia Symbionts Display Variable Infection Patterns That Are Not Predictive of Amoeba Host Outcomes
Source: Genes (Basel). 2020 Jun 20;11(6):674. doi: 10.3390/genes11060674 (PMC7349545; doi:10.3390/genes11060674)

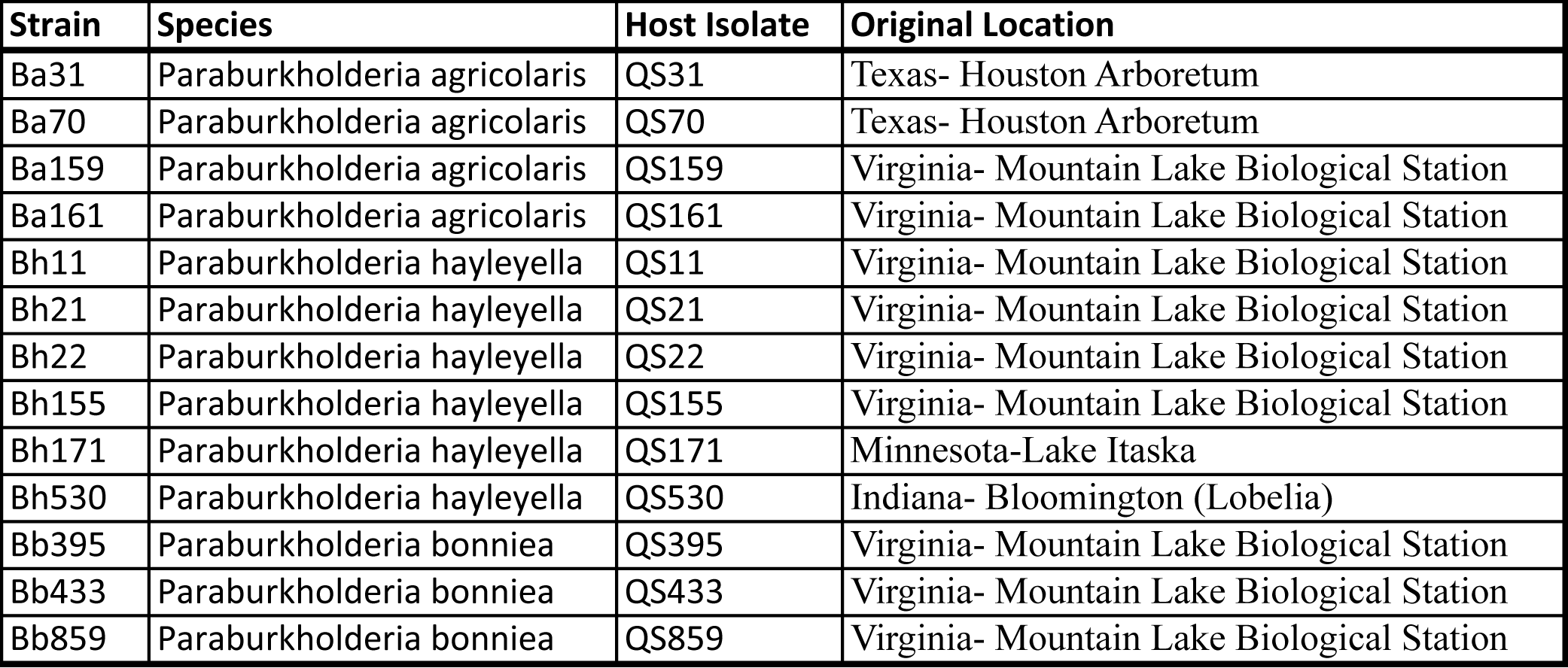

Supplement: Supplementary file 1 [file genes-11-00674-s001.zip › genes-819467-supplementary.tif]
